# Nedd8 hydrolysis by UCH proteases in *Plasmodium* parasites

**DOI:** 10.1371/journal.ppat.1008086

**Published:** 2019-10-28

**Authors:** Maryia Karpiyevich, Sophie Adjalley, Marco Mol, David B. Ascher, Bethany Mason, Gerbrand J. van der Heden van Noort, Heike Laman, Huib Ovaa, Marcus C. S. Lee, Katerina Artavanis-Tsakonas

**Affiliations:** 1 Department of Pathology, University of Cambridge, Cambridge, United Kingdom; 2 Parasites and Microbes Programme, Wellcome Sanger Institute, Cambridge, United Kingdom; 3 Department of Biochemistry, University of Cambridge, Cambridge, United Kingdom; 4 Department of Biochemistry, University of Melbourne, Melbourne, Australia; 5 Oncode Institute and Department of Cell and Chemical Biology, Leiden University Medical Centre, Leiden, The Netherlands; University of South Florida, UNITED STATES

## Abstract

*Plasmodium* parasites are the causative agents of malaria, a disease with wide public health repercussions. Increasing drug resistance and the absence of a vaccine make finding new chemotherapeutic strategies imperative. Components of the ubiquitin and ubiquitin-like pathways have garnered increased attention as novel targets given their necessity to parasite survival. Understanding how these pathways are regulated in *Plasmodium* and identifying differences to the host is paramount to selectively interfering with parasites. Here, we focus on Nedd8 modification in *Plasmodium falciparum*, given its central role to cell division and DNA repair, processes critical to *Plasmodium* parasites given their unusual cell cycle and requirement for refined repair mechanisms. By applying a functional chemical approach, we show that deNeddylation is controlled by a different set of enzymes in the parasite versus the human host. We elucidate the molecular determinants of the unusual dual ubiquitin/Nedd8 recognition by the essential PfUCH37 enzyme and, through parasite transgenics and drug assays, determine that only its ubiquitin activity is critical to parasite survival. Our experiments reveal interesting evolutionary differences in how neddylation is controlled in higher versus lower eukaryotes, and highlight the Nedd8 pathway as worthy of further exploration for therapeutic targeting in antimalarial drug design.

## Author summary

Ubiquitin and ubiquitin-like post-translational modifications are evolutionarily conserved and involved in fundamental cellular processes essential to all eukaryotes. As such, enzymatic components of these pathways present attractive targets for therapeutic intervention for both chronic and communicable diseases. Nedd8 modification of cullin ubiquitin E3 ligases is critical to the viability of eukaryotic organisms and mediates cell cycle progression and DNA damage repair. Given the complex lifecycle and unusual replication mechanisms of the malaria parasite, one would expect neddylation to be of central importance to its survival, yet little is known about this pathway in *Plasmodium*. Here we present our findings on how Nedd8 removal is controlled in *Plasmodium falciparum* and how this pathway differs to that of its human host.

## Introduction

The ubiquitin proteasome system is an ancient and highly conserved pathway essential for the regulation of many important cellular processes in eukaryotes [1]). It is implicated in a great number of diseases, especially cell cycle disorders such as cancer[2–4]. Therapeutic drugs that target the ubiquitin proteasome system are, therefore, in demand with Velcade already being used to treat multiple myeloma patients and several others in clinical trials[5,6]. Ubiquitin is attached to target proteins via an enzyme cascade consisting of an E1 activating enzyme, an E2 conjugating enzyme and an E3 ligase. The number of ubiquitin moieties and the nature of their attachment and branching determines the fate and function of the substrate. This process is reversible and ubiquitin removal is catalyzed by a number of ubiquitin-hydrolases (DUBs). Other ubiquitin-like post-translational modifiers such as SUMO, Nedd8 and Apg8 are conjugated to and released from target proteins following a similar mechanism and using corresponding enzymes.

Infectious pathogens have been found to encode proteins that mimic components of host ubiquitin (Ub) and ubiquitin-like (UBL) cascades. Expression of Ub and UBL enzymes in infected cells disrupts these essential pathways in the host, contributing to immune evasion and manipulation of the cell environment to the pathogen’s advantage[7–9]. Parasitic pathogens, being eukaryotic, have a Ub-proteasome system and Ub-like pathways of their own on which they rely for maintaining homeostasis and survival. Thus, parasite-derived ubiquitin proteasome components may not only mediate key host-pathogen interactions[10,11] but present a potentially potent set of novel drug targets for combating parasitic infections[12,13].

The ubiquitin-proteasome system of *Plasmodium falciparum* has recently garnered increased attention as a target for therapeutic intervention[14]. However, most examples of drugs that target the parasite ubiquitin system are proteasome inhibitors[15,16]. Due to the highly conserved structure of the proteasome, these inhibitors can be non-selective and potentially toxic, although inhibitors with three orders of magnitude preference for the parasite proteasome have been reported[17]. Targeting ubiquitin conjugation and hydrolysis enzymes presents an alternative and promising opportunity to develop inhibitors with potentially greater selectivity or wider applicability. Selective targeting of these enzymes is complicated by their close similarity to host orthologs as is the case with targeting the proteasome. It is therefore critical to identify and exploit structural differences between orthologous enzymes as well as functional differences in how these pathways are regulated differently in parasite and host.

We have previously identified two *P*. *falciparum* (Pf) DUBs, UCHL3 and UCH37 (UCH54)[18,19]. Although *P*. *falciparum* and human UCHL3 share the same specificity, structural analysis revealed distinct differences in the architecture of the active sites that could potentially be exploited to facilitate selective inhibition of the parasite[20]. We also identified the *P*. *falciparum* ortholog of UCH37 using a similar screening methodology but in this case found two notable differences in sequence and in specificity. The *P*. *falciparum* enzyme has a poly-asparagine insertion in the middle of its sequence, increasing the molecular weight to 54 kDa. Furthermore, by reacting with a panel of activity-based probes, we determined this enzyme to have dual Ub/Nedd8 specificity unlike the human ortholog that is solely a DUB[19].

Here we analyze these differences on a molecular basis and elucidate the mechanisms underlying these enzymes’ Nedd8 activity. Nedd8 hydrolysis in mammals is primarily mediated by the CSN5 metalloprotease subunit of the COP9 signalosome[21], which selectively deNeddylates cullin E3 ligases. Processing of the Nedd8 precursor and deNeddylation of non-cullin substrates is mediated by cysteine proteases, including USP21[22], NedP1/Den1/SENP8[23][24][25], and UCHL3[26]. By sequence homology, *Plasmodium* parasites appear to lack a COP9 signalosome and all other known deNeddylases. The one exception is PfUCHL3, an enzyme whose dual Ub/Nedd8 specificity we have previously described[18,20]. Structural studies of human UCHL3[27,28] and PfUCHL3[20] have revealed a cross-over loop that effectively blocks access of larger substrates to the enzyme’s active site[29]. As a result, the biological significance of this enzyme’s activity has been largely assumed to be focused on processing the C-termini of the Nedd8 and Ub precursor proteins rather than contributing to deconjugation of these UBLs from larger substrates. Notably for this study, to date, it has not been possible to uncouple Nedd8 and Ub activities to test their biological relevance separately.

This dearth of predicted deNeddylases suggests that the Nedd8 pathway, despite being evolutionarily highly conserved, may be regulated differently in the malaria parasite versus its mammalian host with potentially novel enzymes mediating its attachment and removal. Herein we use functional activity-based probes to screen for deNeddylating enzymes. We elucidate the molecular determinants of PfUCH37 Nedd8 recognition and successfully uncouple the deNeddylating and deubiquitinating activities of this enzyme. Furthermore, through parasite transgenics, we show that only the PfUCH37 deubiquitinating activity is essential to the viability of blood stage *P*. *falciparum* parasites.

## Results

### *P*. *falciparum* and mammals have different Nedd8 hydrolase repertoires

We have previously identified two dual activity DUB/deNeddylating enzymes in *Plasmodium falciparum* parasites, namely PfUCHL3 and PfUCH37 (also known as PfUCH54)[18,19]. While UCHL3 in both mammals and *Plasmodium* has dual Ub/Nedd8 activity, no UCH37 enzymes, irrespective of organismal origin, have been reported to have Nedd8 activity, making PfUCH37 unique.

By sequence homology, there do not appear to be identifiable *Plasmodium* orthologs to any classical mammalian deNeddylases which suggests that these parasites have a different repertoire of enzymes mediating the Nedd8 pathway.

To capture *Plasmodium*-specific Nedd8-hydrolysing proteins, we screened *P*. *falciparum* lysate with activity-based electrophilic probes. These types of probes have been widely used to identify Ub and other UBL hydrolases[19,30,31]. They comprise a C-terminal electrophilic inhibitor to trap enzymes via their active site cysteine and an N-terminal epitope tag to facilitate retrieval and identification[31]. FLAG-Nedd8-VS, based on the human (Hs) Nedd8 sequence, was reacted with *P*. *falciparum* lysate and probe-enzyme complexes were captured by immunoprecipitation on anti-FLAG resin. Enzymes were identified by LC/MS/MS tandem mass spectrometry, and all protein hits were bioinformatically analysed for presence of defined ubiquitin and ubiquitin-like hydrolytic domains. Only two enzymes were identified with the mammalian probe: PfUCHL3 and PfUCH37 (Table 1).

**Table 1:** DeNeddylating enzymes in *Plasmodium falciparum*.

Protein name	Gene ID	HsNedd8-VS		FLAG-PfNedd8-Prg_1		FLAG-PfNedd8-Prg_2		Activity
		Spectrum count	Coverage %	Spectrum count	Coverage %	Spectrum count	Coverage %	
PfUCHL3	Pf3D7_1460400	1	8.6	5	20.3	0	0	Ub HsNedd8 PfNedd8
PfUCH37	Pf3D7_1117100	36	34.8	15	17.4	5	9.7	Ub HsNedd8 PfNedd8
Ubiquitin carboxyl -terminal hydrolase 13, putative	Pf3D7_0413900	0	0	0	0	4	5.5	Ub
Ubiquitin carboxyl- terminal hydrolase, putative	Pf3D7_0726500	0	0	1	0.8	4	1.9	Unknown (did not express)

*P*. *falciparum* cell lysate was functionally screened for deNeddylases using FLAG-HsNedd8-VS or FLAG-PfNedd8-Prg, the latter was performed twice (FLAG-PfNedd8-Prg_1 and FLAG-PfNedd8-Prg_2). Reactions were followed by anti-FLAG immunoprecipitation and proteomic analysis of bound enzymes. Enzymes were individually expressed in *E*.*coli*, purified, and their specificity was validated by reacting with Ub-AMC, HsNedd8-AMC and PfNedd8-AMC.

Several key differences between *P*. *falciparum* and human Nedd8 made us question whether this probe was indeed capturing all relevant parasite enzymes. Mammalian and *Plasmodium* Nedd8 only share 57% identity (as compared to 99% for ubiquitin) and contain a key difference within their carboxy-termini. The amino acid in position 72 is a part of the C-terminal motif of both ubiquitin and Nedd8 (LXLRGG, where X is amino acid 72), which is very important for the recognition of ubiquitin/Nedd8 by DUBs/deNeddylases and is stabilised by multiple interactions in the protease active site cleft[32,33]. In ubiquitin, amino acid 72 is an arginine (R), in human Nedd8—alanine (A), in *Plasmodium*—glutamine (Q). This variance results in structural differences that are important for substrate selection. Therefore, we repeated the initial screen using an activity based probe derived from the sequence of *P*. *falciparum* Nedd8, FLAG-PfNedd8-Prg. This probe was prepared using solid phase peptide synthesis in analogy to the DUB specific probe, Ub-Prg[34]. This time four enzymes were captured including PfUCHL3 and PfUCH37. The putative enzymes were cloned, recombinantly expressed and their activity was tested *in vitro* using a fluorogenic assay. UBLs modified C-terminally with amidomethyl-coumarin (AMC) release a fluorescent signal when AMC is cleaved by a specific protease. Nedd8-AMC, PfNedd8-AMC and Ub-AMC were all screened. Again, only PfUCH37 (Pf3D7_1117100) and PfUCHL3 (Pf3D7_1460400) were validated as deNeddylases (Table 1) whereas the other two enzymes were determined to have been captured non-specifically. Pf3D7_0413900 was shown to be a strict DUB. Pf3D7_0726500 could not be expressed and tested, however the predicted active site residues appear to be split, potentially only forming a catalytically active enzyme once the protein is folded. We are currently further characterising this protein. All mass spectrometry data are included in S1 Table.

### Multiple sequence alignment reveals clustering of UCH37 orthologs based on Nedd8 activity

**Fig 1:**
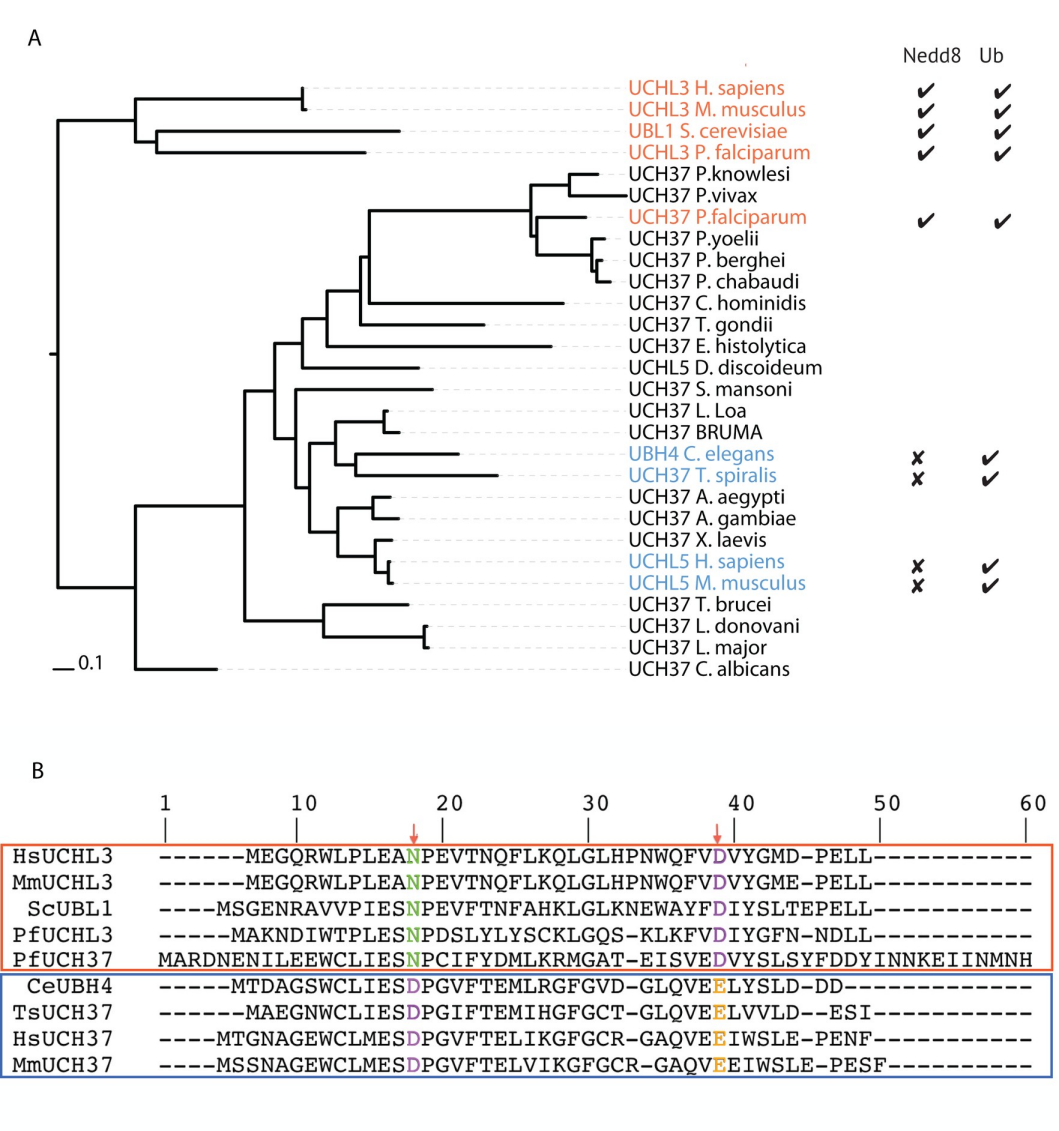
Phylogenetic analysis of UCH37 sequences reveals clustering based on Nedd8 activity. **A)** A dendrogram showing the relative similarity of selected UCH37 enzyme sequences. UCHL3 enzymes with known Nedd8 and Ub specificity are also included for reference. All enzymes with experimentally confirmed Nedd8 activity are colored, with Nedd8-active enzymes highlighted in red and inactive ones in blue. **B)** A multiple alignment of all highlighted enzymes reveals 2 residues (marked with red arrows) whose identity segregates based on Nedd8 activity.

Both PfUCHL3 and PfUCH37 are likely essential to *P*. *falciparum* as determined by the inability to disrupt each gene[20,35]. However, simply knocking out these enzymes would not distinguish whether their DUB or deNeddylating activities are the indispensable function. We, therefore, set out to uncouple these activities to see if we could test them separately. In order to identify specific differences between single and dual activity UCH domain enzymes, we extracted all peptidase C12-containing proteins from protein database Pfam and, using MUSCLE with default parameters[36], aligned them to the UCH37 and UCHL3 enzymes whose activity we had characterized. Using a representative group of enzymes, a dendogram of this alignment was generated (Fig 1A). We cloned, expressed and tested additional orthologs, UBH4 of *C*. *elegans* and that of the nematode *Trichinella spiralis* (TsUCH37)[37]. Both enzymes were Ub-active but neither was able to hydrolyze Nedd8-AMC (S1 Fig). A closer analysis of the alignment revealed patterns of clustering, with two residues (PfUCH37 N18 and D38) conserved amongst the dual activity enzymes versus those with DUB activity alone (Fig 1B). Underscoring the potential importance of these residues to substrate recognition, the crystal structure of the yeast UCH enzyme, YUH-1 with Ub revealed that D35 (corresponding to D38 of PfUCH37) was central to its interaction with Ub[38]. Likewise, a model of PfUCHL3 binding to Nedd8 predicts N13 of PfUCHL3 (N18 of PfUCH37) as being key to their interaction[20].

### Removal of the PfUCH37 poly-asparagine repeat does not inhibit activity

A comparison of human and *P*. *falciparum* UCH37 reveals a low complexity poly-asparagine insertion in the parasite protein that increases its molecular weight to 54kDa (hence we previously referred to it as PfUCH54)[19] (S2A Fig). Poly-asparagine repeats are commonly found in the *Plasmodium* genome, but no specific function has been attributed to them on a transcriptional or a translational level[39]. Previous studies have demonstrated the dispensability of poly-asparagine repeats to protein function[40], and we hypothesized that the same would be the case with PfUCH37. Indeed, deletion of the asparagine-rich stretch (depicted in red) does not affect folding of the catalytic domain nor does it appear to negatively impact enzyme activity against either Ub-AMC or Nedd8-AMC. In fact, the mutant enzyme seemed to be even better at hydrolyzing Nedd8 (S2B Fig). It is possible that the deletion of the poly-asparagine domain may alter some protein dynamics such that Nedd8 binding/release is more efficient. As Nedd8 and Ub have a different surface charge distribution, it could be that the asparagines may interact differently with the two UBLs. However, given that the poly-asparagine domain is unstructured, it is difficult to draw any definitive conclusions. Since we were interested in understanding the molecular basis of this enzyme’s Nedd8 activity, we decided to make use of the deltaN (dN) mutant given its exaggerated phenotype. Thus, any further mention of PfUCH37 in *in vitro* assays refers to the poly-asparagine deletion mutant.

### Ub and Nedd8 activity of PfUCH37 can be uncoupled

We hypothesized that mutating one or both of the highlighted residues in PfUCHL3 (N13D, D33E) and PfUCH37 (N18D, D38E) would abolish Nedd8 activity and that the converse mutations in the human enzyme, HsUCH37 (D12N, E33D), would bring about a gain in deNeddylating activity. Using site-directed mutagenesis we introduced these changes, recombinantly expressed the mutant enzymes and tested their specificity against Ub- and Nedd8-AMC.

The D33E mutation in PfUCHL3 had a mildly inhibitory effect on both Ub-AMC and PfNedd8-AMC processing (S3 Fig). From our previous structural modeling of the PfUCHL3-PfNedd8 complex[20], N13 appeared to have a more central role in stabilising the interaction. However, mutation of N13 to D made the enzyme slightly more active against both Ub- and Nedd8-AMC (S3 Fig). Notably, neither mutation on it own nor both together affected either Ub or Nedd8 hydrolysis selectively. We performed extensive *in silico* modeling whereby the predicted binding affinity of PfUCHL3 to Ub and Nedd8 was assessed for every possible substitution of each amino acid at the interacting interface. Aside from the N13D mutation already tested *in vitro*, this analysis did not reveal any further residues expected to differentially affect the enzyme’s Nedd8 and Ub activities (S2 and S3 Tables).

Likewise, the corresponding PfUCH37 N18D mutation left the Ub and Nedd8 activities of the enzyme largely unaffected (S4 Fig). This mutation was not predicted to alter interactions within the structures of the complexes, nor was it predicted to alter the affinity of the complexes by the protein-protein interaction analysis tool, mCSM-PPI[41].

**Fig 2:**
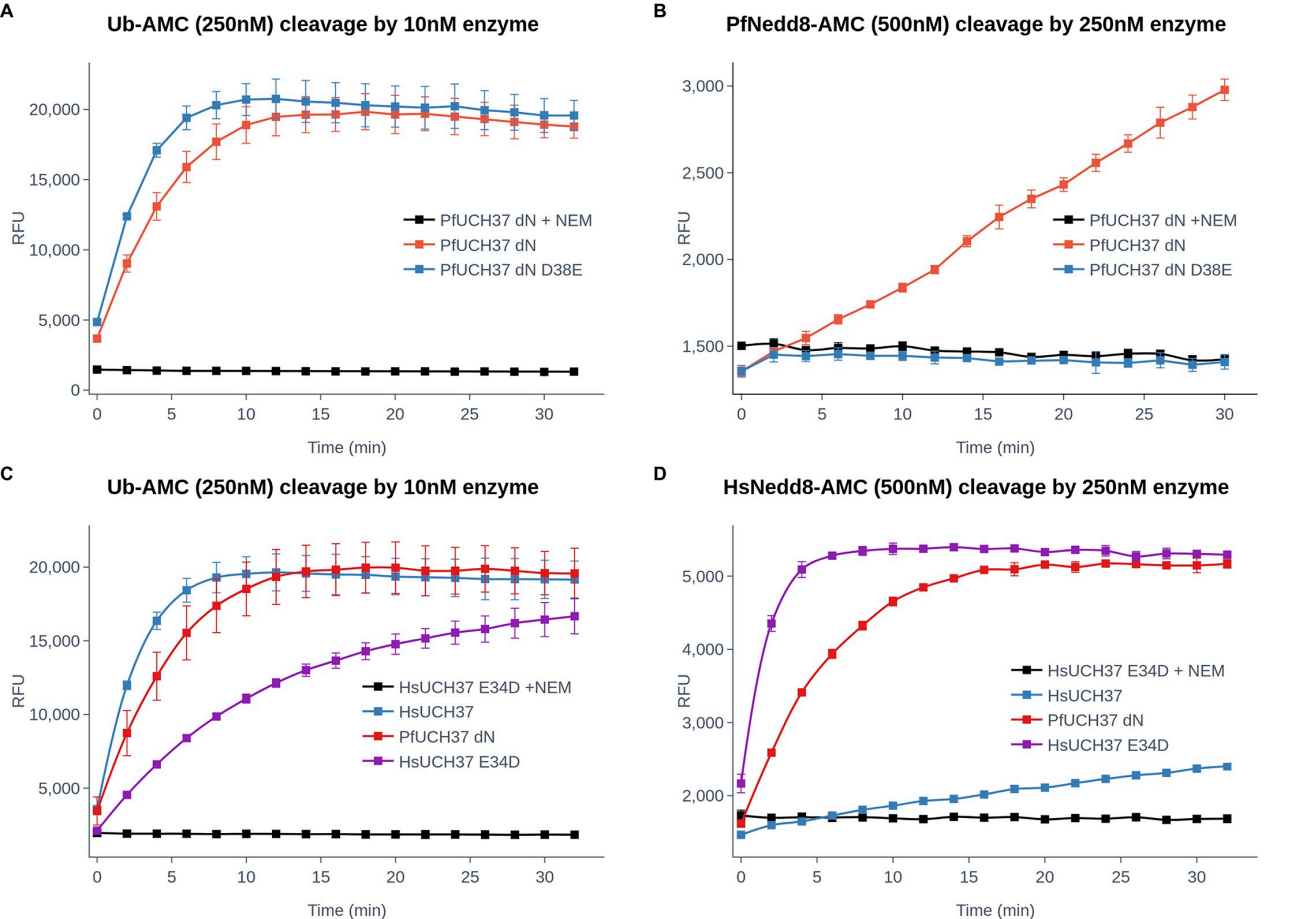
Ubiquitin and Nedd8 enzyme activity can be uncoupled. Enzymatic activity of PfUCH37 and HsUCH37 wild type and mutant enzymes was tested by Ub-AMC and Nedd8-AMC hydrolysis. A Ub-AMC assay was done using recombinant **A)** PfUCH37 dN and a D38E mutant or **C)** HsUCH37 and its E34D mutant. 10nM of enzyme was incubated with an excess of Ub-AMC (250 nM) and hydrolysis was measured in relative fluorescence units. PfNedd8-AMC hydrolysis by **B)** PfUCH37 dN and its D38E mutant or **D)** HsUCH37 and its E34D mutant, was measured using 250nM of recombinant protein incubated with 500 nM of PfNedd8-AMC. Cleavage was measured by fluorescence output every 15 seconds for a minimum of 30 minutes and as a negative control, enzymes were pre-incubated with NEM for 15 minutes prior to being used in the assays. Error bars correspond to standard deviation from triplicate repeats.

However, when we replaced the PfUCH37 D38 with E, we saw a striking effect, namely the near elimination of Nedd8 activity while hydrolysis of Ub-AMC remained unaffected (Fig 2A and 2B). Notably, the reverse mutation in the human UCH37 turned the enzyme into one whose Ub proteolysis is significantly decreased and Nedd8 proteolysis is dramatically increased, resulting in a mutant with both functions (Fig 2C and 2D). We confirmed this phenomenon by introducing the corresponding mutation in TsUCH37 (E32D) and testing its Nedd8 activity with similar results (S1D Fig). Thus, the substitution of a single amino acid succeeded in uncoupling the Ub and Nedd8 specificity of these enzymes.

**Fig 3:**
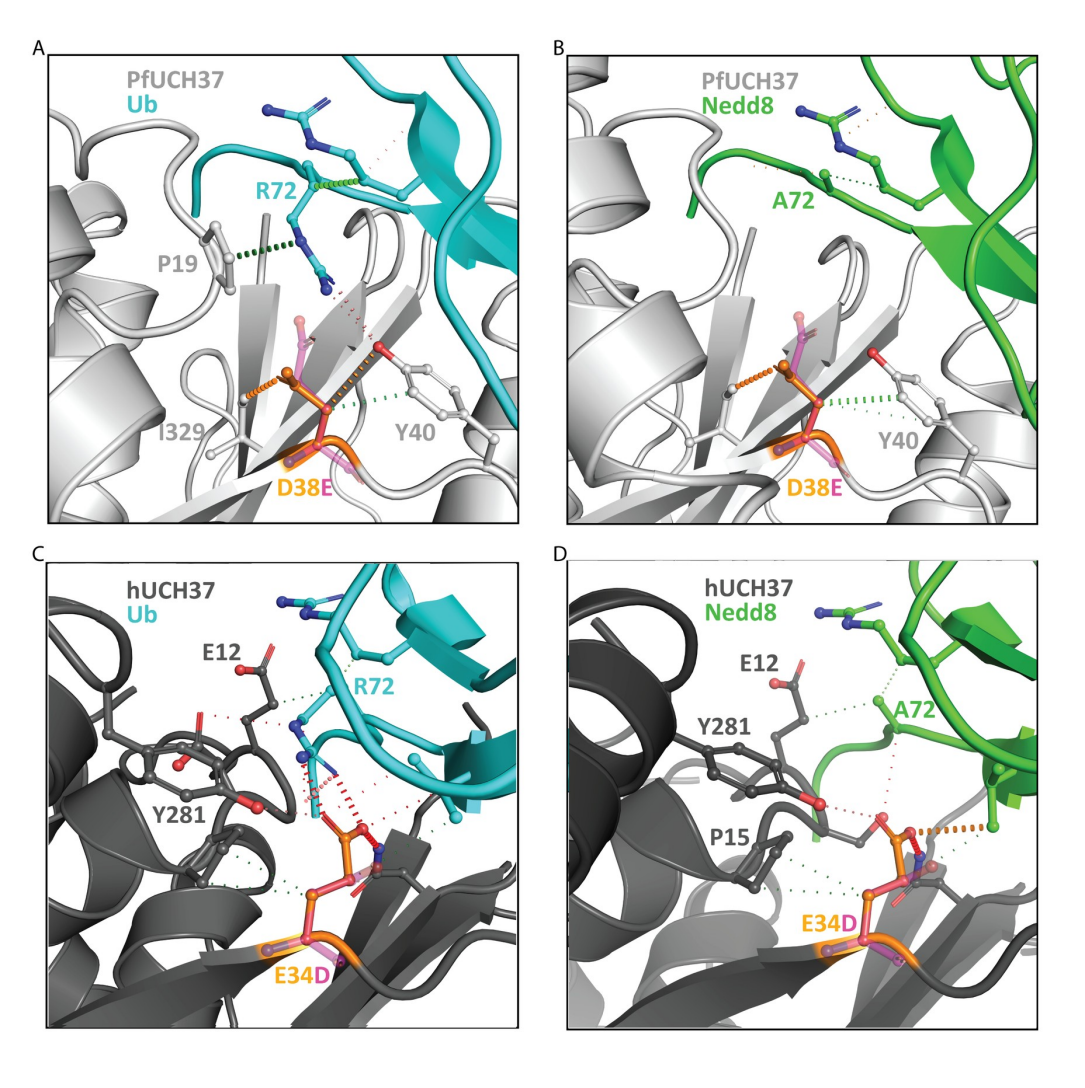
Structural models showing the effects of the mutations on the ubiquitin and Nedd8 binding pocket of UCH37 enzymes. Ribbon representations of PfUCH37 (light grey) and HsUCH37 (dark grey) in complex with **A)** and **C)** human ubiquitin (cyan) and **B)** and **D)** HsNedd8 (green). The wild type UCH37 residues are shown in orange, and the mutant in magenta. Polar interactions are shown in orange, hydrogen bonds in red and hydrophobic interactions in green.

In order to understand the conformational change a D versus an E might impart on the overall protein as well as its interaction with UBLs, we generated structural models of wild type and mutant PfUCH37 and HsUCH37 in complex with both Nedd8 and ubiquitin using the experimental crystal structures of related UCH37 enzymes (Fig 3).

In the structures of PfUCH37 and HsUCH37, D38 and E34, respectively, are located in a groove at the Ub/Nedd8 binding interface, making strong inter-molecular interactions that drive binding affinity and specificity to Ub and Nedd8 (Fig 3). These residues are located near residue 72 of ubiquitin (R72) and Nedd8 (A72), key residues that have been previously shown to modulate E1 selectivity specificity[33]. Binding to this region stabilizes the enzyme-substrate complex and positions the cleavage site relative to the catalytic site of UCH37. Within PfUCH37, the mutation D38E would recreate the formation of the hydrogen bonds to R72 in ubiquitin seen in the crystal structure of the complex with HsUCH37. By contrast, the extension of the carboxylic acid moiety of the glutamic acid into the binding pocket would alter this interaction with the distal Nedd8, such that the stabilised conformation no longer promotes the exo-specific cleavage of Nedd8 by UCH37. This was consistent with the mCSM-PPI predictions that indicated that mutation of D38E in PfUCH37 would slightly improve binding to ubiquitin, but significantly destabilise binding to Nedd8.

In HsUCH37, E34 is positioned to make key hydrogen bonds to R72 of ubiquitin. Upon mutation to D, these interactions would be lost; however, additional interactions to D12 and Y262 of ubiquitin could be formed without altering the orientation of the stabilised distal ubiquitin. By contrast, this E34D mutation would open up the binding pocket to allow binding of Nedd8. This was consistent with the mCSM-PPI predictions that E34D would improve binding to Nedd8 but have minimal effect on ubiquitin recognition.

### PfUCH37 Nedd8 activity is dispensable to parasite viability

**Fig 4:**
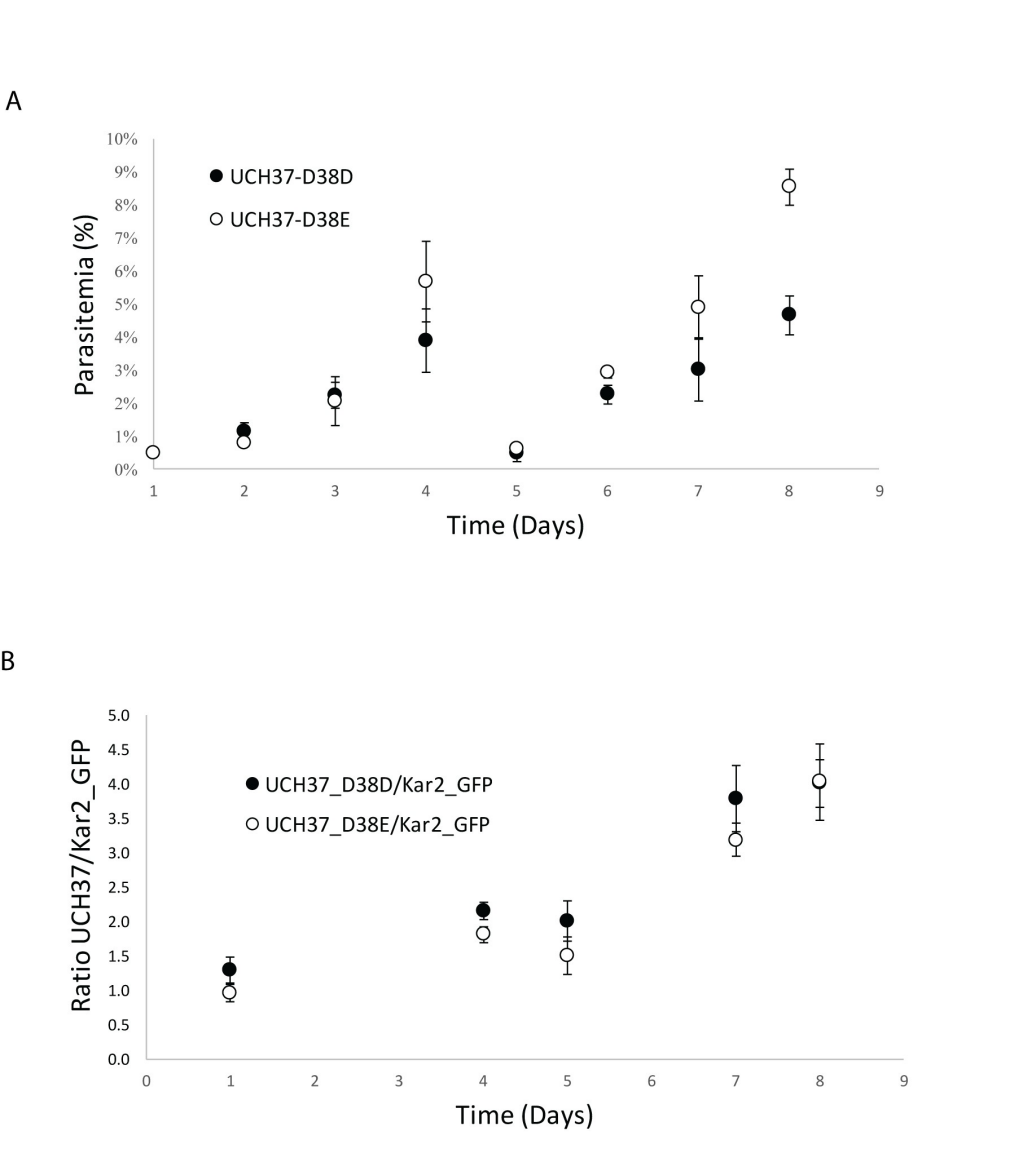
D38E mutation in PfUCH37 does not affect parasite fitness. *P*. *falciparum* Dd2 parasites were edited using CRISPR/Cas9 to generate PfUCH37 D38E mutants or PfUCH37 D38D silent mutants as controls. **A)** Parasite growth was assessed over the course of 8 days, with parasitemia recorded on a daily basis for both parasite lines. **B)** A head-to-head competition assay was done to measure growth of each mutant line against the Kar2_GFP reference line over the course of 8 days. Data points are expressed as ratios (D38E:GFP and D38D:GFP). Error bars correspond to standard deviation from triplicate repeats.

**Fig 5:**
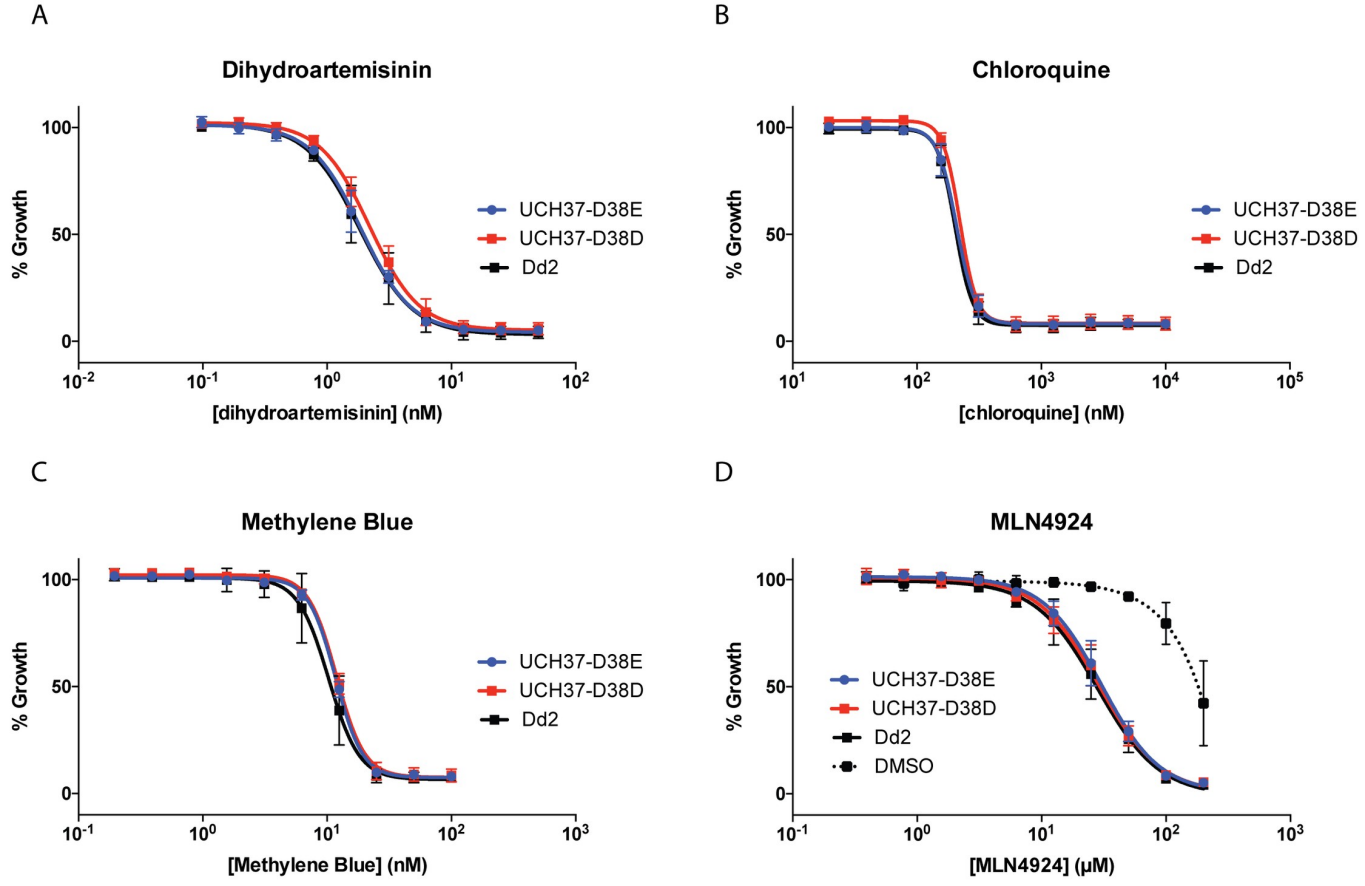
PfUCH37 D38E mutants and wild type parasites respond similarly to drug challenge. PfUCH37 D38E mutants, D38D controls and the Dd2 wild type parent line were treated for 72-h with a 2-fold serial dilution of **A)** dihydroartemisinin (from 50 nM to 0.1 nM), **B)** chloroquine (10 μM to 0.02 μM), **C)** methylene blue (100 nM to 0.2 nM) or D) MLN4924 (200 μM to 0.391 μM) and parasite growth measured by SYBR green assay. In panel **D**, the dotted line corresponds to the equivalent concentration of DMSO, which at the highest concentration used (1% v/v) has an impact on parasite growth. Data reported as means ± standard errors with n≥ 3.

In mice, disruption of UCH37 is embryonic lethal[42]. Assuming evolutionary conservation, one would expect its ubiquitin activity to be central to parasite survival as well. Indeed, in a large-scale insertion mutagenesis screen in *P*. *falciparum*, no insertions were observed in PfUCH37, suggesting essentiality[35]. Similar genetic studies in the mouse malaria parasite, *P*. *berghei*, suggest that parasite growth is significantly impaired in its absence[43]. However, functional disruption does not address whether the Ub or Nedd8 activity is responsible for this phenotype. Considering the absence of other classical deNeddylases, we hypothesized that PfUCH37 should regulate deNeddylation in *P*. *falciparum* and that this activity should be essential. With our newfound ability to uncouple the two activities, we used CRISPR/Cas9 to edit the endogenous gene to introduce the D38E mutation. As a control, a second parasite line was generated for which only silent mutations were introduced (D38D). Clonal lines were isolated by limiting dilution, and were used to examine the phenotypic effects on parasite growth, as well as sensitivity to cell stress induced by the antimalarials dihydroartemisinin (DHA), chloroquine and methylene blue. Contrary to our expectation, the mutant parasites grew normally (Fig 4). When comparing the D38E to the D38D lines over the course of 8 days, the D38E parasites appeared to have accelerated growth at higher parasitemias (Fig 4A). However, when each mutant line was subjected to a head-to-head competition assay against a GFP reference line, there was no significant difference in their rates of growth (Fig 4B). Furthermore, the D38E parasites did not display increased sensitivity to drug treatment (Fig 5A–5C). When treated with the human Nedd8 E1 inhibitor, MLN4924, both mutant and wild type parasites succumbed equally at 10μM concentrations and above (Fig 5D). This result further underscores the irrelevance of PfUCH37 Nedd8 activity to parasite viability, however it also indicates that a functional Nedd8 pathway is essential.

There is precedent in the literature for disruption of deNeddylation having no overt phenotype: Nedd8 is essential to *S*. *pombe* fission yeast, however simultaneous knockout of all enzymes with Nedd8-hydrolysing activity does not affect growth and viability[44]. Although deletion of NedP1 in the mammalian system is also not lethal, NedP1 -/- cells are reported to have altered responses to oxidative stress[45,46]. However, since artemisinin is known to affect parasites through induction of oxidative stress and since we saw no change in sensitivity to DHA, this mechanisms is unlikely to occur in the PfUCH37 D38E line.

### *Plasmodium* UCHL3 cannot deNeddylate Cullin-1

Given that we only found two deNeddylases in our screen and determined the activity of PfUCH37 to be non-essential, we wondered if PfUCHL3 is, in fact, capable of mediating Nedd8 removal from protein substrates. Although our previous data would lead us to expect that the deNeddylating activity of PfUCHL3 is limited to processing the Nedd8 precursor protein, it has more recently been shown that mammalian UCH family enzymes are indeed capable of processing Ub attached to larger proteins as long as the protein in question is not another Ub[47]. These data would suggest that the inability of UCHL3 to process polyUb chains is not a result of steric hindrance to its active site, but rather something to do with the chemistry of the Ub-Ub interaction. Given this enzyme’s high affinity to human Nedd8-AMC, we tested whether it would be capable of deNeddylating an active (neddylated) Cullin-1 RING E3 ligase complex immunoprecipitated out of HEK293T cells (S5A Fig). Both the neddylated and unneddylated forms of Cullin-1 were detectable by blot. Immunoprecipitated protein was treated with purified PfUCHL3, PfUCH37dN or human COP9 signalosome as a positive control, separated on SDS-PAGE and probed with an anti-Cullin-1 antibody to assess presence or absence of the neddylated form. Despite being capable of hydrolysing human Nedd8-AMC, neither PfUCHL3 nor PfUCH37dN demonstrated activity against neddylated human Cullin-1 (S5B Fig). Given the mixture of parasite enzymes and human Cullin-1, we still cannot exclude the possibility that these *P*. *falciparum* enzymes might be active against physiological PfNedd8-modified substrates. Once neddylated substrates are identified *in vivo*, we will be able to definitively assess this point.

## Discussion

Protein neddylation is an essential process in a wide range of organisms including mammals[48], plants[49], lower eukaryotes[50,51] and yeast[52]. Although a comprehensive functional analysis of this pathway in parasitic systems is ongoing, orthologs of Nedd8, the E1, E2 and E3 enzymes involved in its conjugation, and its best-defined substrate, the cullin E3 ligases, can all be identified in these organisms [12,53]. In *P*. *falciparum*, the presence of putative Nedd8, the catalytic subunit of the Nedd8 E1 (UBA3) and the Nedd8 E2 (UBC12) ligases suggests that this pathway is indeed functional. Moreover, UBA3 and UBC12 are both nonmutable genes[35], indicating that neddylation is an essential process. The absence of NedP1, USP21 and the COP9 signalosome, inclusive of the CSN5 metalloprotease subunit, suggests alternate mechanisms of deNeddylation control in these parasites. Screening *P*. *falciparum* lysate with Nedd8 activity-based probes identified only two potential candidates: PfUCHL3 and PfUCH37 ubiquitin hydrolases, both likely essential enzymes[20][35]. UCH enzymes are characterized by an active site cross-over loop. UCHL3 has a tighter, more restrictive loop[27] that extends across the C-terminal residue of the Ub moiety when bound[28,38]. This conformation requires that substrates be of sufficiently small size to pass through[29] or that bonds to be hydrolysed have specific characteristics[47]. As a result, the physiological functions of UCHL3 are assumed to be centered around processing Ub and possibly Nedd8 precursors. The cross-over loop of UCH37, however, is expanded and capable of accommodating larger substrates including ubiquitin chains, particularly when its C-terminal tail extension is bound by the proteasome (or other physiological partners) and catalytic activity is maximized[54]. We would expect that in the presence of a physiological substrate that binds its C-terminal tail, the efficiency of PfUCH37 Nedd8 deconjugation would be even more appreciable than what we observed *in vitro*. Therefore, it is conceivable that PfUCH37 can deNeddylate substrates normally relegated to NedP1 control[25,33].

By successfully uncoupling the Ub and Nedd8 activity, we were able to demonstrate that PfUCH37-mediated deNeddylation is dispensable, at least as pertains to the asexual blood-stage. It will be interesting to assess whether these mutant parasites are disadvantaged at other developmental stages or more susceptible to certain stress stimuli. Experiments are underway to test these possibilities. Why *Plasmodium* would retain an enzyme with a seemingly irrelevant specificity is puzzling. Furthermore, how its Nedd8 hydrolysis is regulated, be it by localization, protein interactions or other means is also of interest.

Although we were unable to uncouple Nedd8 and Ub activities of PfUCHL3, we are inclined to believe that this enzyme is unlikely to be the main deNeddylase in *Plasmodium* parasites given its inability to process neddylated Cullin-1. The absence of other deNeddylases in our screen begs the questions of how the Nedd8 pathway is controlled in parasites, which proteins are the target substrates and whether Nedd8 hydrolysis is even essential. Considering the probes we used in our screens are specific to cysteine proteases and that Cullins are exclusively deNeddylated by the CSN5 metalloprotease subunit of the COP9 signalosome (absent in *P*. *falciparum*), it is possible that an unidentified, parasite-specific metalloprotease is fulfilling this function. However, searching PlasmoDB for the InterPro (www.ebi.ac.uk/interpro/) domain IPR000555 (JAB1/MPN/MOV34 metalloenzyme domain) returns only three proteins in the *P*. *falciparum* proteome. Two of them are the orthologs of the proteasome subunits Rpn11 and Rpn8, while the third one is a putative eukaryotic translation initiation factor 3 subunit f (eIF3f). Although PfRpn11 contains an active MPN+ domain, PfRpn8 and PfeIF3f have inactive MPN- domains that lack the essential catalytic residues.

Taking the D/E dichotomy as predictive of Nedd8 specificity, an alignment of all members of the peptidase C12 family (including all UCHL3 and UCHL5/37 enzymes) identifies mostly budding yeast and fungi as having a dual specificity UCH enzyme. This is not surprising since, like *S*. *cerevisiae* Yuh-1, these organisms possess a single UCH enzyme. However, two non-plasmodial parasites are predicted to have a dual activity UCH37: the Chinese liver fluke, *Clonorchis sinensis* and all species of the *Leishmania* genus. Notably, *C*. *sinensis* also has an ortholog of NedP1 whereas the *Leishmania* species do not. Given the recent sequencing of many parasitic and metazoan genomes, it will be interesting to perform a more detailed analysis across eukaryotes to assess whether UCH37 dual specificity and NedP1 presence are somehow functionally linked and to assess whether additional factors might influence the behavior of this enzyme *in vivo*.

In conclusion, our findings raise interesting points regarding the evolution of neddylation, highlighting differences within this essential pathway between *P*. *falciparum* and the human host. We are currently working towards fully characterising this pathway in the parasite to determine whether these differences can be targeted for the development of novel therapeutics against malaria.

## Materials and methods

### Cloning, mutagenesis and protein expression

PfUCH37 (PfUCH54) and PfUCHL3 were previously cloned into a bacterial expression vector pET28a+[19][20]. Other UCH enzymes were amplified from cDNA or genomic DNA of respective organism and cloned into pet28a+. PfUCH37dN was created by removal of the poly-asparagine (polyN) domain (amino acids 251–298 of PfUCHL3) using PCR-mediated deletion. Site-directed mutagenesis to generate PfUCH37 D38E, HsUCH37 E34D, TsUCH37 E32D, PfUCHL3 N13D, PfUCHL3 D33E, and PfUCHL3 N13D D33E was done using the Agilent Site-Directed Mutagenesis Kit following manufacturer’s specifications. BL21-CodonPlus (DE3)-RIL *E*. *coli* (Agilent) were used for protein expression. Bacterial cultures were grown at 37°C, and protein expression was induced with 0.1 mM IPTG at OD_600_ of 0.6 for 18 hours at 18°C. Soluble proteins were purified via their 6HIS tag on NiNTA resin (Qiagen) following manufacturer’s specifications.

### Probe generation and screening

*Plasmodium falciparum* 3D7 parasites were cultured as previously described[55]. Schizont cell pellets were lysed in lysis buffer (50 mM Tris, 150 mM NaCl, 1% Triton X-100, 5mM L-cysteine) on ice and centrifuged at 13.3 krpm for 15 minutes. 1mM FLAG-NEDD8-VS (BostonBiochem) or FLAG-PfNedd8-Prg (prepared using solid phase peptide synthesis as described in Ekkebus *et al*.[34]) was added to the cell lysate supernatants and incubated for 90 minutes at 37°C. FLAG fusion proteins were then immunoprecipitated using Anti-FLAG M2 Affinity Gel (Sigma-Aldrich) and eluted using 3X FLAG peptide (Sigma-Aldrich) according to the manufacturer’s instructions. NuPAGE 4–12% Bis-Tris gels were used for SDS-PAGE of the eluates, followed by staining with Coomassie Blue.

### Mass spectrometry analysis

1D gel bands were excised and transferred into a 96-well PCR plate. The gel bands were cut into 1mm^2^ pieces, destained, reduced (DTT) and alkylated (iodoacetamide) and subjected to enzymatic digestion with trypsin overnight at 37°C. After digestion, the supernatant was pipetted into a sample vial and loaded into an autosampler for automated LC-MS/MS analysis.

LC-MS/MS experiments were either performed using a Dionex Ultimate 3000 RSLC nanoUPLC (Thermo Fisher Scientific Inc, Waltham, MA, USA) system coupled with an Orbitrap Lumos mass spectrometer (Thermo Fisher Scientific Inc, Waltham, MA, USA) or a nanoAcquity UPLC (Waters Corp., Milford, MA) system coupled to an LTQ Orbitrap Velos mass spectrometer (Thermo Scientific, Waltham, MA). In both cases, separation of peptides was performed using C18 reverse-phase chromatography at a flow rate of 300 nL/min and a total LC run time of 60 minutes. In each case, CID MS/MS spectra of eluting peptides were collected automatically by means of linear ion trap mass analysers.

Post-run, all MS/MS data were converted to mgf files and the files were then submitted to the Mascot search algorithm (Matrix Science, London UK) and searched against the Uniprot Plasmodium falciparum database (UP000001450) and a common contaminant sequences containing non-specific proteins such as keratins and trypsin (115 sequences, 38274 residues). Variable modifications of oxidation (M) and deamidation (NQ) were applied as well a fixed modification of carbamidomethyl (C). The peptide and fragment mass tolerances were set to 25ppm and 0.8 Da, respectively. A significance threshold value of p<0.05 and a peptide cut-off score of 20 were also applied. All data was then imported into the Scaffold program (Version_4.5.4, Proteome Software Inc, Portland, OR).

### AMC assays

Proteins were used in Ub-AMC and Nedd-AMC (Boston Biochem) assays as previously described[56]. Briefly, enzyme concentrations were adjusted using BCA assay (Pierce), and reactions were initiated by addition of Ub- or Nedd8-AMC substrate at the indicated concentrations. Fluorescence was measured in relative fluorescence units (RFU) on a BMG FLUOstar Omega plate reader.

### Parasite mutagenesis by CRISPR/Cas9 editing

Mutant PfUCH37-D38E and silent control PfUCH37-D38D parasites were generated using CRISPR/Cas9. A guide RNA (TATGATATGCTTAAACGTAT) targeting a site in PfUCH37 25 bp upstream of the D38 codon was designed using Benchling (https://benchling.com/). Complementary oligos encoding the gRNA sequence (tattTATGATATGCTTAAACGTAT / aaacATACGTTTAAGCATATCATA) were phosphorylated using T4 polynucleotide kinase (5U for 30 min at 37˚C) and annealed (95˚C for 5 min, followed by step down of 5˚C/min to 25˚C). The oligo mixture was diluted 1/200 in water and ligated into the BbsI-digested pDC2-coCas9-U6.2-hDHFR plasmid[57]. Donor templates (390 bp in length, starting 76 bp upstream of the UCH37 coding sequence) were synthesized as GeneStrings by ThermoFisher and inserted into the AatII/EcoRI sites of the pDC2-coCas9-U6.2-hDHFR plasmid by Gibson assembly, to generate the final transfection vectors, pDC2-coCas9-UCH37-D38E-gRNA-hDHFR and pDC2-coCas9-UCH37-D38D-gRNA-hDHFR.

Ring-stage parasites (Dd2) at 5% parasitemia were transfected by electroporation with a BioRad Gene Pulser II at 310V and 950μF with 50μg of each plasmid DNA. Transformed parasites were then selected for 10 days with 2.5 nM WR99210 (Jacobus Pharmaceuticals), followed by culturing in complete media without drug selection. Edited clones were then isolated by serial limiting dilution and confirmed by Sanger sequencing after PCR amplification from genomic DNA using forward primer 5’ CCTTGCGAAAAAATGTATTATTTCA and reverse primer 5’ GGAAACAAAATGAAAGGAGTCT.

### Parasite growth and drug sensitivity assays

Dd2 parasites were propagated as previously described[55] in RPMI-1640 with 50 mg/l hypoxanthine, 0.25% sodium bicarbonate, 0.5% Albumax II and 25 mM HEPES (Life Technologies) and supplemented with GlutaMAX at 1x concentration (Gibco). To evaluate the fitness of the PfUCH37-modified parasites, we measured parasite growth using two different methods. Overall growth of the PfUCH37-D38E and D38D control lines was measured by flow cytometry (Beckman Coulter Cytoflex) on a daily basis, with an initial starting parasitemia of 0.5%. Upon crossing the 5% parasitemia threshold, parasitemia was restored to 0.5% to prevent overgrowth and stress. A second method to assess relative growth rates was performed by a competitive growth assay with a fluorescent reference line[58]. Test parasites (either PfUCH37-D38E or PfUCH37-D38D) were mixed in a 1:1 ratio with the fluorescent reference line, Dd2-GFP, in which an integrated eGFP reporter was expressed from the strong ER hsp70 (PF3D7_0917900) promoter. At regular intervals an aliquot of each culture was first stained with Mitotracker Far Red, and the ratio of the GFP-negative test line to the GFP-positive reference was measured by flow cytometry. Note that due to slower growth of the reference line due to high levels of GFP overexpression, the test lines eventually overgrow the reference line.

To assess their effect on PfUCH37-modified parasites, a two-fold serial dilution of each compound (MLN4924, Bio-Techne; dihydroartemisinin, Cambridge Bioscience; chloroquine and methylene blue, Sigma-Aldrich) was performed in technical duplicate using 96-well flat bottom plates. Parasites at predominantly ring-stage were added at 1% parasitemia (1% final haematocrit). Plates were incubated at 37˚C for 72 hours and parasite growth measured using SYBR green I stain (Life Technologies) prepared in lysis buffer (final concentration 1x SYBR green I stain in 5 mM Tris-HCl, 1.5 mM EDTA, 0.05% w/v saponin, 0.5% v/v Triton X-100), incubated at 37˚C for 30 minutes, and fluorescence signal measured on a FLUOstar Omega (BMG Biotech). At least three independent experiments were performed to test parasite drug sensitivity.

### Purification of SCF^Fbxl17^ complexes and *in vitro* deNeddylation

HEK293T cells (ATCC) were transfected with SCF components (Skp1, Cul1, Myc-Rbx1) and FLAG-Fbxl17. After 48 hours, cells were resuspended in lysis buffer (LB) (25 mM Tris-HCl, pH 7.5, 225 mM KCl, 1% NP-40) with a protease inhibitor cocktail (Sigma-Aldrich) and phosphatase inhibitors (10 mM NaF, 1 mM PMSF, 1mM Na_3_VO_4_). Lysates were incubated with Anti-FLAG M2 Affinity Gel (Sigma-Aldrich) for 5 hours at 4°C with rotation. Beads were washed in LB and eluted in 300 μg/mL FLAG peptide (Sigma-Aldrich) in elution buffer (10 mM HEPES, pH 7.9, 225 mM KCl, 1.5 mM MgCl_2_, 0.1% NP-40) for 1hr at 4°C with rotation. Purified 500nM SCF complex (wt) was incubated with 100nM PfUCHL3, PfUCH37dN or human COP9 signalosome (Enzo, BML-PW9425) for 2.5 hours at 37°C (in 25mM Tris pH7.4, 100mM NaCl, 1mM DTT and 5% glycerol), followed by SDS-PAGE and Western blots. Anti-Cullin-1 (abcam, ab75812, 1:1000), anti-Nedd8 (GeneTex, GTX61205, 1:1000) Anti-Skp1 (BD Biosciences, 610530, 1:1000), anti-myc-tag (Rbx1), anti-myc (Cell Signalling, 2272, 1:1000), anti-polyhistidine (Sigma, H1029, 1:2000) and anti-CSN5/JAB1 (Novus Biologicals, 2A10, 1:1000) primary antibodies were used for Western blots.

### Structural modeling

Molecular models were generated using Modeller[59] and MacroModel (Schrodinger, New York, NY). PfUCH37 was modelled using an ensemble of available UCH37 X-ray crystal structures (PDB IDs: 3A7S and 3IHR, 44% and 36% sequence identity respectively), including the complex of TsUCH37 with ubiquitin (37% sequence identity; PDB ID: 4IG7). The structures of PfUCH37 and TsUCH37 (PDB ID: 4IG7) were minimized using the MMF94s forcefield in Sybyl-X 2.1.1 (Certara L.P., St Louis, MO), with the final structure having more than 95% of residues in the allowed region of a Ramachandran plot. Ubiquitin and Nedd8 were docked into the structures of UCH37 using Piper (Schrodinger, New York, NY), with the available X-ray crystal structure of TsUCH37 in complex with ubiquitin (PDB ID: 4IG7) used to guide protein docking. The models of the complexes were minimised using the MMF94s forcefield in Sybyl-X 2.1.1 as described above. The quality of all the models were confirmed with Verify3D[60]. The structural consequences of the differences in interfacial residues were analysed to assess the structural importance of the residues[41,61–63]. Interactions were calculated using Arpeggio[64] and model structures were examined using Pymol.

## Supporting information

S1 Table Mass spectrometry data for activity-based probe pulldowns. Table summarizing the results of the three pulldown experiments performed (PfNedd8-Prg twice and HsNedd8-VS once) with protein IDs and spectral counts. Peptide identities are included in the second tab and ones matching to hits included in Table 1 are highlighted in yellow.
(XLSX)

S2 Table Heat map of residues predicted to change PfUCHL3-Ub, -PfNedd8 and -HsNedd8 binding affinities. The heat map plots the change in binding affinity upon mutation (as the change in the Gibb’s free energy of binding) between PfUCHL3 and Ub (top), PfNedd8 (middle) and HsNedd8 (bottom). A negative value is predictive of a mutation expected to disrupt the interaction, whereas a positive value is predictive of a mutation expected to stabilise the interaction.
(PDF)

S3 Table Heatmap of PfUCHL3 residues expected to affect Ub and Nedd8 binding affinities differently. To identify mutations in PfUCHL3 predicted to have a different effect upon Ub and Nedd8 binding, changes in binding affinity upon mutation (as the change in the Gibb’s free energy of binding) have been calculated as differences between HsNedd8 and Ub (top) versus PfNedd8 and Ub (bottom). Differences have been expressed as Nedd8 –Ub so that mutations predicted to affect Nedd8 binding more than Ub binding are negative values (red); and those predicted to affect Ub binding more than Nedd8 binding are positive (blue).
(PDF)

S1 Fig T. spiralis UCH37 and C. elegans UCH37 orthologs are DUBs but not deNeddylases. Enzymatic activity of TsUCH37 and CeUBH4 (the UCH37 ortholog) was tested by Ub-AMC and Nedd8-AMC hydrolysis. A Ub-AMC assay was done using recombinant **A)** CeUBH4 or **C)** TsUCH37. Enzyme at the indicated concentrations was incubated with an excess of Ub-AMC (250 nM) and hydrolysis was measured in relative fluorescence units. Nedd8-AMC hydrolysis by **B)** CeUBH4 or **D)** TsUCH37 and its E32D mutant, was measured using recombinant protein at the indicated concentration incubated with 500 nM of Nedd8-AMC. Cleavage was measured by fluorescence output every 15 seconds for a minimum of 30 minutes and as a negative control, enzymes were pre-incubated with NEM for 15 minutes prior to being used in the assays. Error bars correspond to standard deviation from triplicate repeats.
(PDF)

S2 Fig PfUCH37dN retains the ability to hydrolyse Nedd8 and Ubiquitin AMC substrates. **A)** Human and *P*. *falciparum* UCH37 protein sequences were aligned using MUSCLE. The polyasparagine repeat in PfUCH37 is highlighted in red and catalytic residues in blue. **B)** Ub-AMC Assays were done using recombinant PfUCH37wt and PfUCH37dN proteins. 250nM of protein was incubated with an excess of Ub-AMC (250 nM) to measure the ability to hydrolyze Ub-AMC conjugate. PfNedd8-AMC assays were completed using 250nM of recombinant protein incubated with 250 nM of PfNedd8-AMC. Cleavage was measured by fluorescence output every 15 seconds for a minimum of 30 minutes. Error bars correspond to standard deviation from triplicate repeats.
(PDF)

S3 Fig N13D and D33E mutation do not affect PfUCHL3 deNeddylating activity. Enzymatic activity of PfUCHL3 wild type and mutant enzymes was tested by Ub-AMC and Nedd8-AMC hydrolysis. Ub-AMC **A)** and PfNedd8-AMC **B)** assays were done using recombinant wild type PfUCHL3, a N13D mutant, a D33E mutant and a double mutant. Enzymes at the specified concentrations were incubated with an excess of substrate and hydrolysis was measured in relative fluorescence units every 15 seconds for a minimum of 30 minutes. As a negative control, wild type enzyme was pre-incubated with NEM for 15 minutes prior to being used in the assays. Error bars correspond to standard deviation from triplicate repeats.
(PDF)

S4 Fig N18D mutation does not uncouple PfUCH37 DUB and deNeddylating activities. Enzymatic activity of PfUCH37 wild type and N18D mutant enzymes was tested by Ub-AMC and Nedd8-AMC hydrolysis. A **A)** Ub-AMC assay and a **B)** PfNedd8-AMC assay were done using recombinant PfUCH37 wild type enzyme and a N18D mutant on the same background. Enzyme at the indicated concentrations was incubated with an excess of Ub-AMC (250 nM) or Pf-Nedd8-AMC 2.5uM) and clevage was measured by fluorescence output every 15 seconds for a minimum of 30 minutes and as a negative control, PfUCH37 N18D was pre-incubated with NEM for 15 minutes prior to being used in the assays. Error bars correspond to standard deviation from triplicate repeats.
(PDF)

S5 Fig PfUCHL3 cannot deNeddylate Cullin-1. SCF components (Skp1, Cul1, Myc-Rbx1) and FLAG-Fbxl17 (wt or ΔFbox) were co-immunoprecipitated out of HEK293T using anti-FLAG resin and presence of each component was verified by immunoblot **(A)**. The ability of recombinant HIS-PfUCHL3 and HIS-PfUCH37dN to cleave HsNedd8 off of Cullin-1 was assessed by anti-Cul1 and anti-Nedd8 immunoblot **(B)**. PfUCHL3 and PfUCH37dN were detected by anti-HIS and COP9 was probed by anti-CSN5 (the catalytic component of the COP9 signalosome) immunoblot.
(PDF)
