# Supplementary figures and images for: Nedd8 hydrolysis by UCH proteases in Plasmodium parasites
Source: PLoS Pathog. 2019 Oct 28;15(10):e1008086. doi: 10.1371/journal.ppat.1008086 (PMC6837540; doi:10.1371/journal.ppat.1008086)

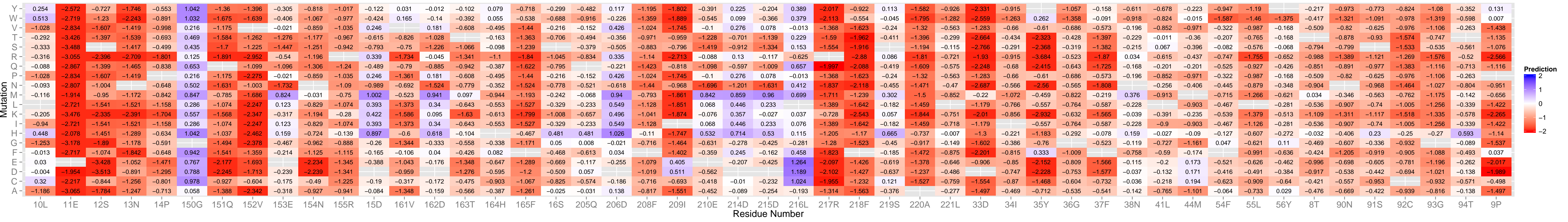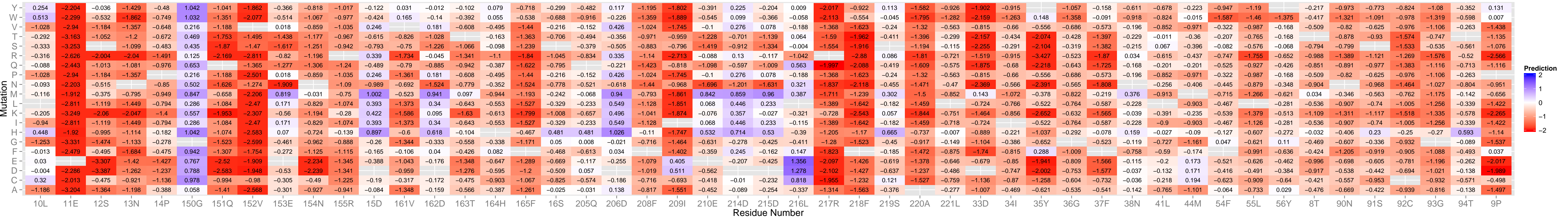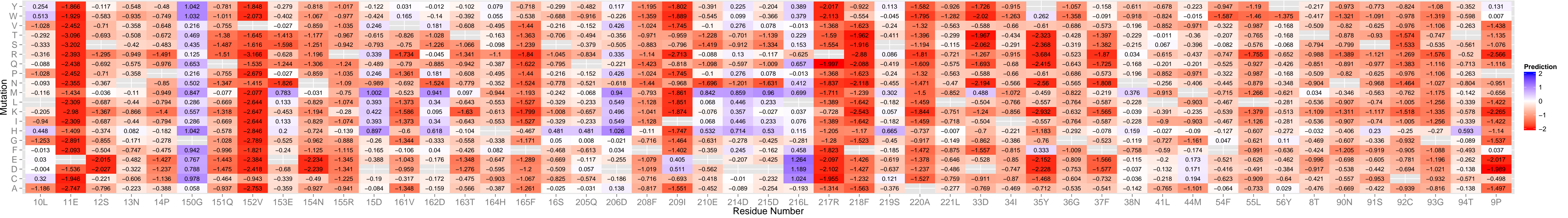

Supplement: S2 Table — The heat map plots the change in binding affinity upon mutation (as the change in the Gibb’s free energy of binding) between PfUCHL3 and Ub (top), PfNedd8 (middle) and HsNedd8 (bottom). A negative value is predictive of a mutation expected to disrupt the interaction, whereas a positive value is predictive of a mutation expected to stabilise the interaction. (PDF) [file ppat.1008086.s002.pdf]

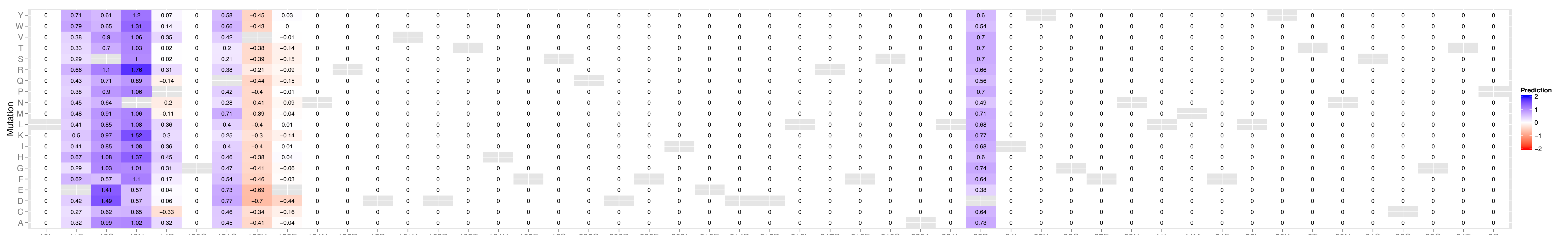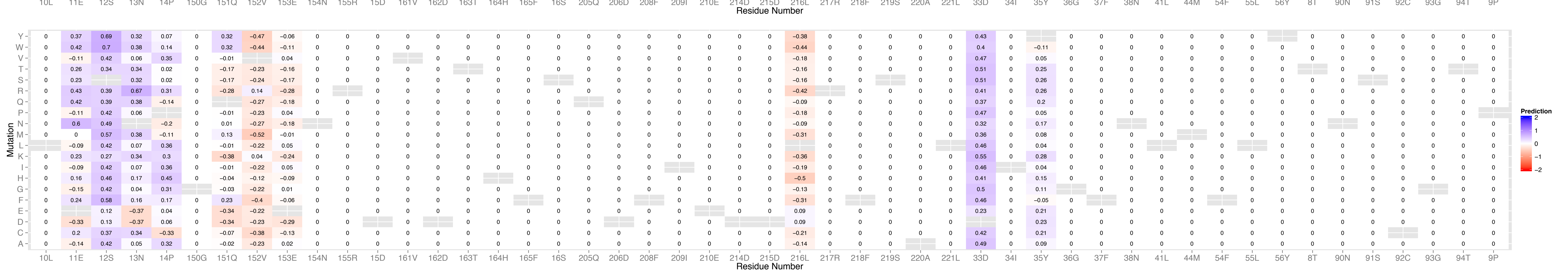

Supplement: S3 Table — To identify mutations in PfUCHL3 predicted to have a different effect upon Ub and Nedd8 binding, changes in binding affinity upon mutation (as the change in the Gibb’s free energy of binding) have been calculated as differences between HsNedd8 and Ub (top) versus PfNedd8 and Ub (bottom). Differences have been expressed as Nedd8 –Ub so that mutations predicted to affect Nedd8 binding more than Ub binding are negative values (red); and those predicted to affect Ub binding more than Nedd8 binding are positive (blue). (PDF) [file ppat.1008086.s003.pdf]

Supp Figure 1

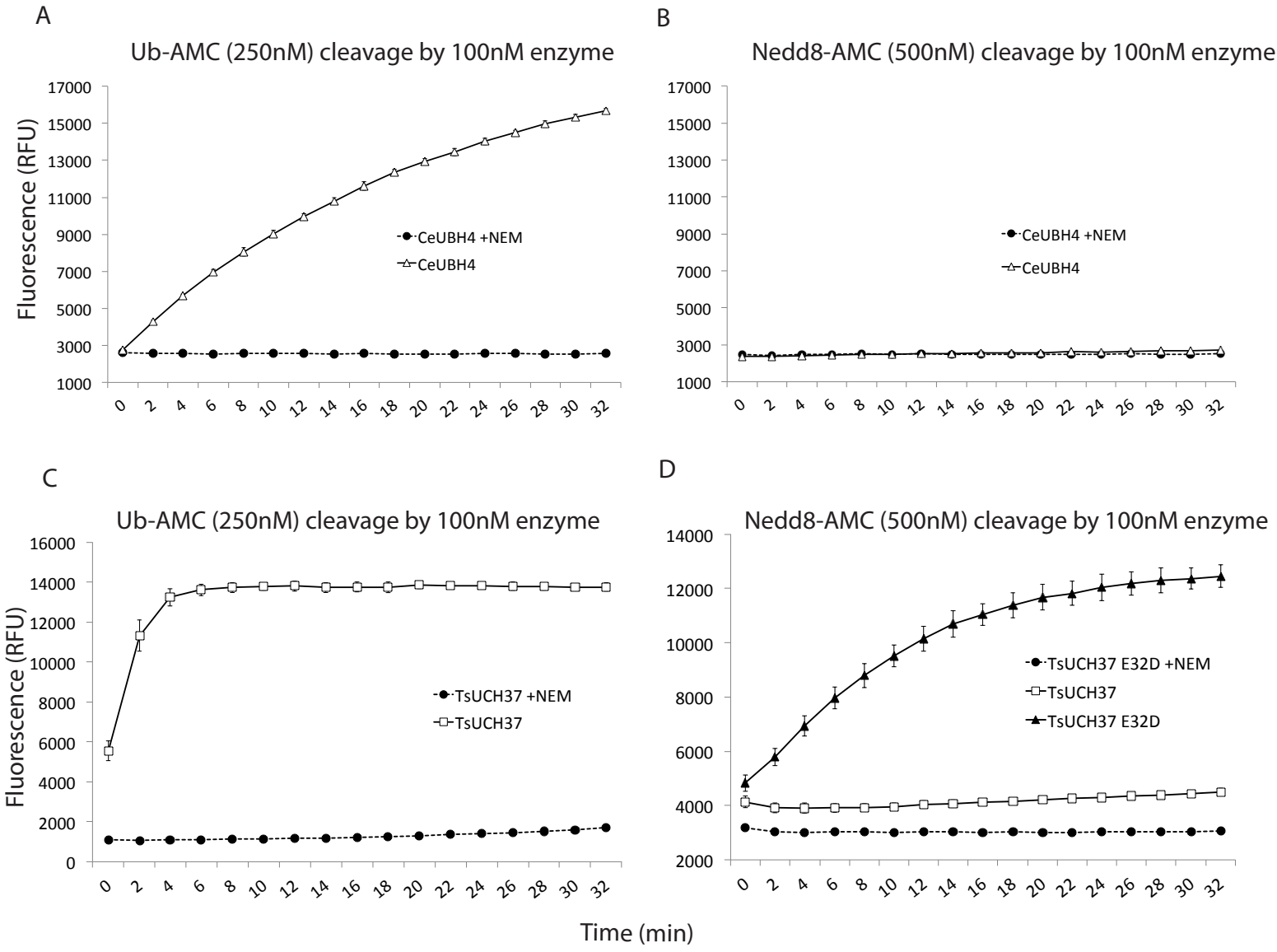

Supplement: S1 Fig — Enzymatic activity of TsUCH37 and CeUBH4 (the UCH37 ortholog) was tested by Ub-AMC and Nedd8-AMC hydrolysis. A Ub-AMC assay was done using recombinant A) CeUBH4 or C) TsUCH37. Enzyme at the indicated concentrations was incubated with an excess of Ub-AMC (250 nM) and hydrolysis was measured in relative fluorescence units. Nedd8-AMC hydrolysis by B) CeUBH4 or D) TsUCH37 and its E32D mutant, was measured using recombinant protein at the indicated concentration incubated with 500 nM of Nedd8-AMC. Cleavage was measured by fluorescence output every 15 seconds for a minimum of 30 minutes and as a negative control, enzymes were pre-incubated with NEM for 15 minutes prior to being used in the assays. Error bars correspond to standard deviation from triplicate repeats. (PDF) [file ppat.1008086.s004.pdf]

Supplementary Figure 3

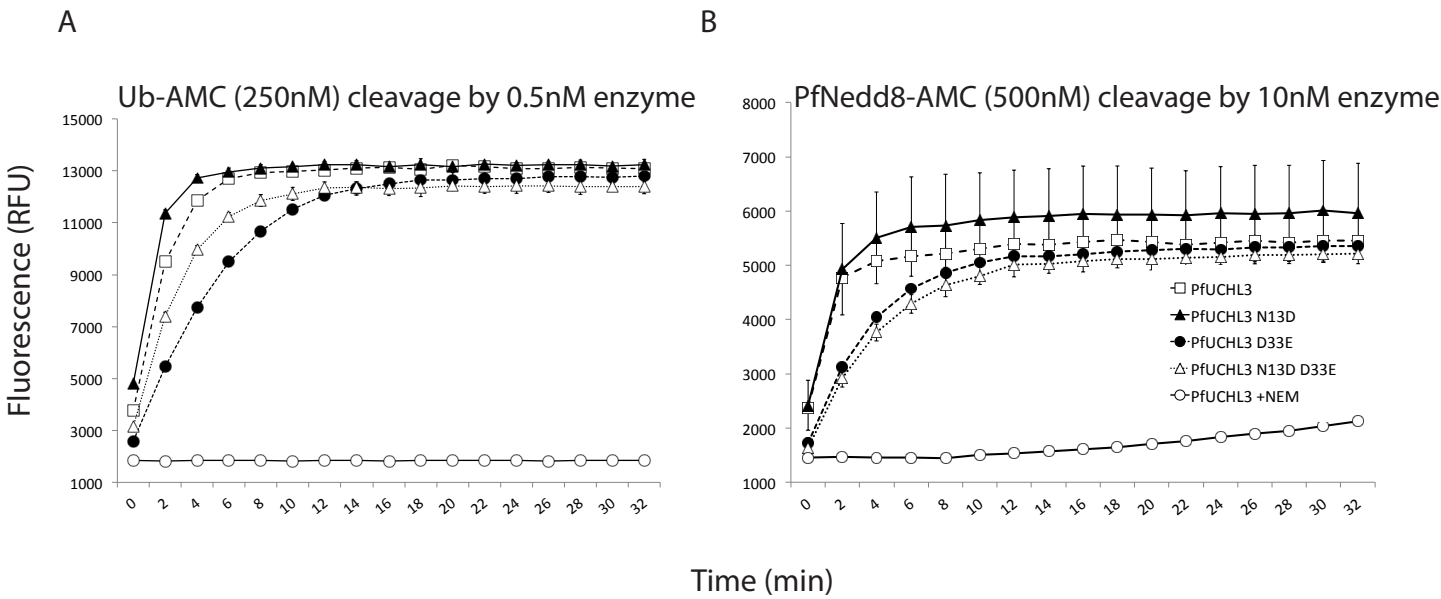

Supplement: S3 Fig — Enzymatic activity of PfUCHL3 wild type and mutant enzymes was tested by Ub-AMC and Nedd8-AMC hydrolysis. Ub-AMC A) and PfNedd8-AMC B) assays were done using recombinant wild type PfUCHL3, a N13D mutant, a D33E mutant and a double mutant. Enzymes at the specified concentrations were incubated with an excess of substrate and hydrolysis was measured in relative fluorescence units every 15 seconds for a minimum of 30 minutes. As a negative control, wild type enzyme was pre-incubated with NEM for 15 minutes prior to being used in the assays. Error bars correspond to standard deviation from triplicate repeats. (PDF) [file ppat.1008086.s006.pdf]

Supp Figure 4

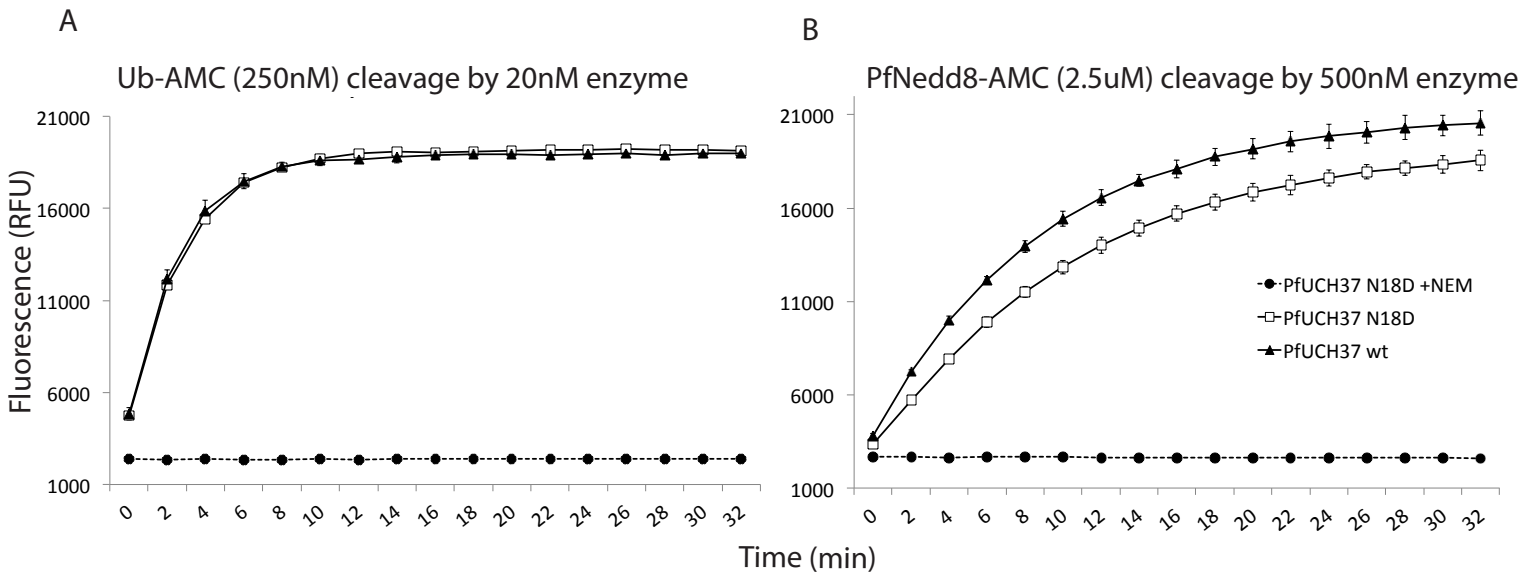

Supplement: S4 Fig — Enzymatic activity of PfUCH37 wild type and N18D mutant enzymes was tested by Ub-AMC and Nedd8-AMC hydrolysis. A A) Ub-AMC assay and a B) PfNedd8-AMC assay were done using recombinant PfUCH37 wild type enzyme and a N18D mutant on the same background. Enzyme at the indicated concentrations was incubated with an excess of Ub-AMC (250 nM) or Pf-Nedd8-AMC 2.5uM) and clevage was measured by fluorescence output every 15 seconds for a minimum of 30 minutes and as a negative control, PfUCH37 N18D was pre-incubated with NEM for 15 minutes prior to being used in the assays. Error bars correspond to standard deviation from triplicate repeats. (PDF) [file ppat.1008086.s007.pdf]

Supp Figure 5

A

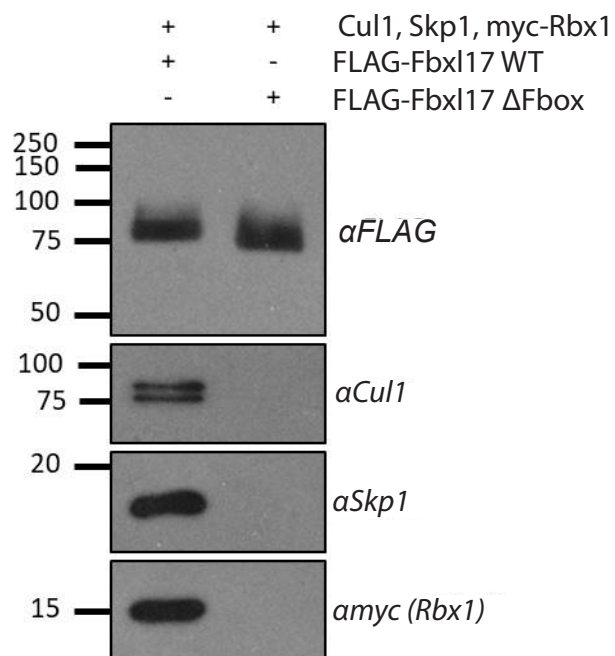

B

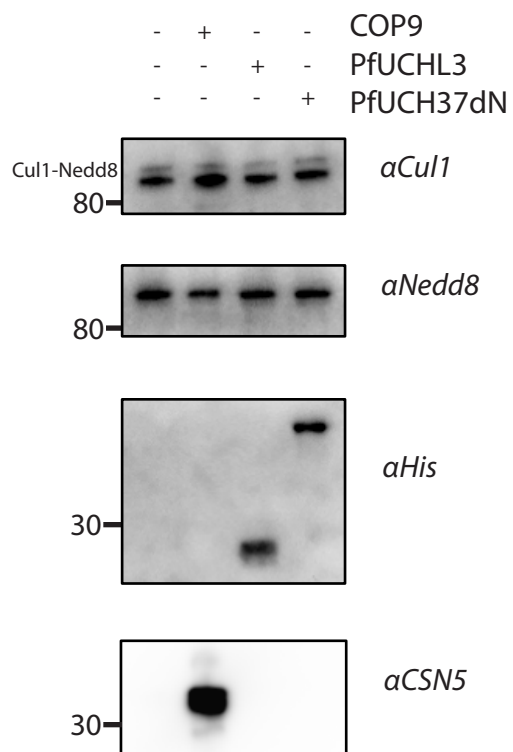

Supplement: S5 Fig — SCF components (Skp1, Cul1, Myc-Rbx1) and FLAG-Fbxl17 (wt or ΔFbox) were co-immunoprecipitated out of HEK293T using anti-FLAG resin and presence of each component was verified by immunoblot (A). The ability of recombinant HIS-PfUCHL3 and HIS-PfUCH37dN to cleave HsNedd8 off of Cullin-1 was assessed by anti-Cul1 and anti-Nedd8 immunoblot (B). PfUCHL3 and PfUCH37dN were detected by anti-HIS and COP9 was probed by anti-CSN5 (the catalytic component of the COP9 signalosome) immunoblot. (PDF) [file ppat.1008086.s008.pdf]
