# Supplementary material for: Nedd8 hydrolysis by UCH proteases in Plasmodium parasites
Source: PLoS Pathog. 2019 Oct 28;15(10):e1008086. doi: 10.1371/journal.ppat.1008086 (PMC6837540; doi:10.1371/journal.ppat.1008086)

Supp Figure 2

A

```
HsUCH37   MTGNA----GEWCLMESDPGVFTELIKGFGCRGAQVEEIWSLE-----------------  39
PfUCH37   MARDNENILEEWCLIESNPCIFYDMLKRMGATEISVEDVYSLSYFDDYINNKEIINMNHI  60

HsUCH37   ---------------------------------PENFEKLKP----VHGLIFLFKWQPGE  62
PfUCH37   LGVDTYLGENNKTLDKENNVVDVIELYKNNICMEDKYNKLLKHHSYIYGIIFLFNIGKHY 120

HsUCH37   EPAGSVVQDSRLDTIFFAKQVINNACATQAIVSVLLNCTHQDVHLGETLSEFKEFSQSFD 122
PfUCH37   K-NNKYIEHNVPDNLFFAKQVIPNACATQAILSIVLN---KDIELNDEIKNIKTFSLNFD 176

HsUCH37   AAMKGLALSNSDVIRQVHNSFARQQMFEFDT--KTSAKEEDAFHFVSYVPVNGRLYELDG 180
PfUCH37   SSMKGLTLSNCTFLRNIHNSYKPPIYLDKEDVHHDKKKSEDSFHFVSYISFQDKVYLLDG 236

HsUCH37   LREGPIDLGACNQD---------------------------------------------- 194
PfUCH37   LQSGPVLINADEQNKPNPNNNNNNKDNDNDNNNNNNNNNNNNNNNNNNNNNNNNNNIGMN 296

HsUCH37   --DWISAVRPVIEKRIQKY----SEGEIRFNLMAIVSDRKMIYEQKIA------------ 236
PfUCH37   GKDWIEISREHIKKEIDEICNSQTNNDVRFNIIAVMKDKEYIIQEYINIHRIVKQRVNIK 356

HsUCH37   --------ELQRQLAEEE-------------PMDTDQGNSMLSAIQSEVAKNQMLIEEEV 275
PfUCH37   LINLGENIELSDEINEDEFPLLNDIPSIENLPNNVDTLYNIVNKSTLEINYLQSLLHEQK 416

HsUCH37   QKLKRYKIENIRRKHNYLPFIMELLKTLAEHQQLIPLVEKAKEKQNAKKAQETK 329
PfUCH37   EIKKLWNKELTFKFFNFYPFIMSSLNLMAKHK----LLKDAYQKEKLKNATKS- 465
```

B

Ub-AMC (250nM) cleavage by 250nM enzyme

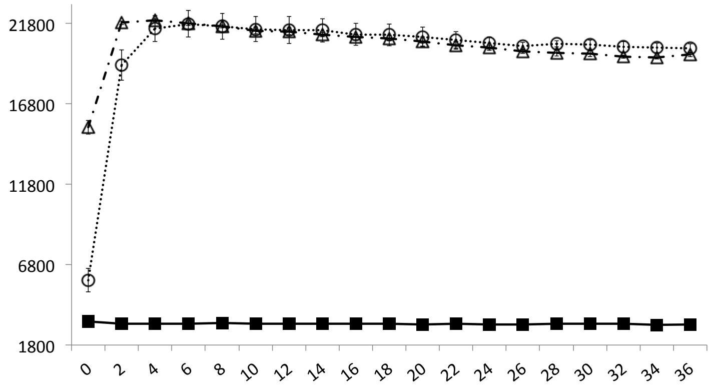

PfNedd8-AMC (250nM) cleavage by 250nM enzyme

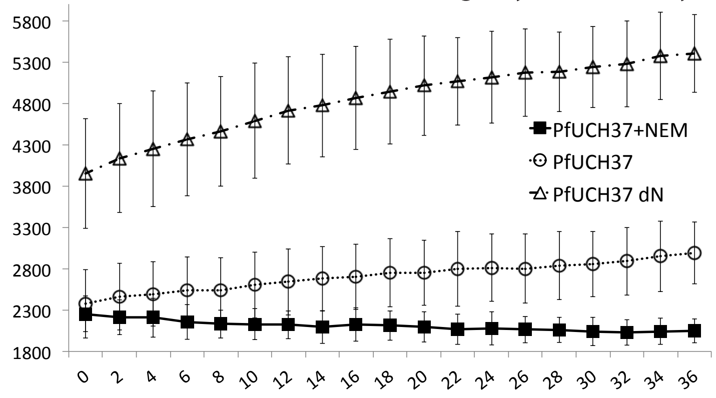

Supplement: S2 Fig — A) Human and P. falciparum UCH37 protein sequences were aligned using MUSCLE. The polyasparagine repeat in PfUCH37 is highlighted in red and catalytic residues in blue. B) Ub-AMC Assays were done using recombinant PfUCH37wt and PfUCH37dN proteins. 250nM of protein was incubated with an excess of Ub-AMC (250 nM) to measure the ability to hydrolyze Ub-AMC conjugate. PfNedd8-AMC assays were completed using 250nM of recombinant protein incubated with 250 nM of PfNedd8-AMC. Cleavage was measured by fluorescence output every 15 seconds for a minimum of 30 minutes. Error bars correspond to standard deviation from triplicate repeats. (PDF) [file ppat.1008086.s005.pdf]
